# Advancing Total Synthesis Through Skeletal Editing

**DOI:** 10.1021/acs.accounts.5c00030

**Published:** 2025-04-10

**Authors:** Reem Al-Ahmad, Mingji Dai

**Affiliations:** Department of Chemistry, Emory University, Atlanta, Georgia 30322, United States

## Abstract

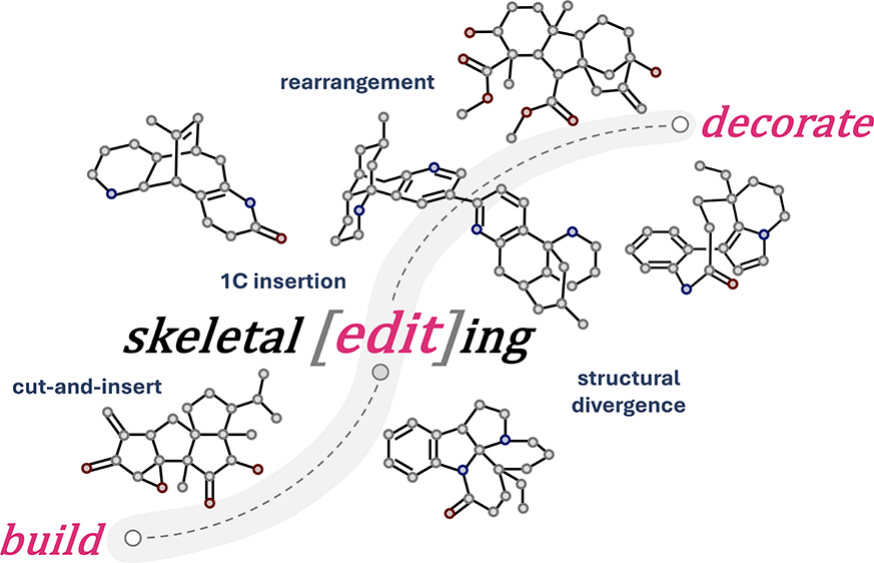

Total synthesis has long been a proving ground for advancing chemical thought, pushing chemists to develop strategies that not only replicate nature’s complexity but often surpass it. The pursuit of efficiency, practicality, and elegance continues to challenge and reshape the guiding principles of total synthesis. In recent years, skeletal editing has emerged as a powerful strategy for reconfiguring skeletal frameworks in ways that were previously difficult to imagine. Unlike conventional chemical synthesis approaches, which primarily rely on the logic of bond construction reactions and functional group manipulations, skeletal editing introduces elements that allow for atom insertion, deletion, and exchange and skeletal rearrangement/reorganization by harnessing the potential energy and reactivity of certain structural motifs and morphing them into new electronic and spatial configurations. The logic of modern skeletal editing has been fueling the development of new editing methods and advancing the fields of total synthesis, medicinal chemistry, materials science, and others.

In this Account, we detail our program using skeletal editing-based retrosynthetic logic to facilitate natural product synthesis. We first highlight two one-carbon insertion editing strategies utilizing the Ciamician–Dennstedt rearrangement and the Büchner–Curtius–Schlotterbeck ring expansion to streamline the total syntheses of complanadine and phleghenrine *Lycopodium* alkaloids. We next present our synthesis of crinipellin and gibberellin diterpenes by leveraging the facile synthesis and intrinsic strain of cyclobutanes as precursors to challenging cyclopentanes via cut-and-insert editing (crinipellins) or C–C bond migratory ring expansion (GA_18_). Toward the end, we describe our early efforts in orchestrating structural rearrangement and functional group pairing reactions to access seven monoterpene indole alkaloids and highlight the divergent potential of skeletal editing. Each of the five examples follows a build–edit–decorate workflow, inspired by Schreiber’s build–couple–pair in diversity-oriented synthesis. In the build stage, key scaffolds are efficiently assembled from starting materials with matched reactivity. The edit stage morphs these scaffolds to the desired but more challenging ones encoded by the target molecules, reminiscent of Corey’s application of rearrangement transforms as a topological strategy. The decorate stage introduces additional functional groups and adjusts oxidation states to complete the total synthesis, similar to the oxidase phase of Baran’s two-phase synthesis. The essence of skeletal editing-based retrosynthetic analysis is to identify latent structural relationships between the readily assembled key scaffolds constructed in the build stage and the desired ones encoded by the target molecules as well as proper editing methods to transform the former into the latter with precision. The build–edit–decorate approach parallels the dynamism of biosynthesis, enabling rapid building of complexity with great efficiency and step economy, as analyzed by the spacial scores (SPS) of each case. Drawing on these principles, chemists can adopt skeletal editing-based retrosynthetic logic by identifying latent intermediates and employing and developing strategic editing methods to overcome synthetic bottlenecks.

## Key References

- MaD.; MartinB. S.; GallagherK. S.; SaitoT.; DaiM.One-Carbon Insertion and Polarity Inversion Enabled a Pyrrole Strategy to the Total Syntheses of Pyridine-Containing Lycopodium Alkaloids: Complanadine A and Lycodine. J. Am. Chem. Soc.2021, 143, 16383–1638734570487 10.1021/jacs.1c08626PMC9123642.^1^*This work demonstrates a pyrrole-to-pyridine one-carbon insertion skeletal editing strategy for total syntheses of Lycopodium alkaloids complanadine A and lycodine and provides a new retrosynthetic logic for complex pyridine-containing natural products and beyond*.
- CaiX.; LiL.; WangY.-C.; ZhouJ.; DaiM.Total Syntheses of Phleghenrines A and C. Org. Lett.2023, 25, 5258–526137432129 10.1021/acs.orglett.3c01784PMC10367062.^2^*This work demonstrates how a retrosynthetic one-carbon deletion can significantly simplify the total synthesis of phleghenrine alkaloids by harnessing the power of Diels–Alder cycloadditions and one-carbon insertion*.
- XuB.; ZhangZ.; TantilloD. J.; DaiM.Concise Total Syntheses of (−)-Crinipellins A and B Enabled by a Controlled Cargill Rearrangement. J. Am. Chem. Soc.2024, 146, 21250–2125639052841 10.1021/jacs.4c07900PMC11311239.^3^*This work demonstrates a cut-and-insert editing process via a controlled Cargill rearrangement in accessing the tetraquinane crinipellins and highlights the efficiency of photochemical [2 + 2] cycloaddition in building congested ring systems and all-carbon quaternary centers*.
- LiL.; LiangW.; RiveraM. E.; WangY.-C.; DaiM.Concise Synthesis of (−)-GA_18_ Methyl Ester. J. Am. Chem. Soc.2023, 145, 53–5736573889 10.1021/jacs.2c12470PMC11167720.^4^*This work demonstrates how strategic deployment of photochemical [2 + 2] cycloaddition and skeletal rearrangement can rapidly assemble challenging trans-hydrindane and bicyclo[3.2.1]octane scaffolds and facilitate efficient access to gibberellin diterpenoid*.
- YangY.; BaiY.; SunS.; DaiM.Biosynthetically Inspired Divergent Approach to Monoterpene Indole Alkaloids: Total Synthesis of Mersicarpine, Leuconodines B and D, Leuconoxine, Melodinine E, Leuconolam, and Rhazinilam. Org. Lett.2014, 16, 6216–621925412144 10.1021/ol503150cPMC4260631.^5^*This work demonstrates the divergent potential of skeletal editing to access structurally diverse alkaloids via selective bond scission and reorganization and functional group pairing*.

## Introduction

The synthesis of urea by Wöhler in 1828 marked the birth of the total synthesis of naturally occurring molecules. The discipline now is approaching its bicentennial and its mission continues to evolve.^6^ Total synthesis has long been used to confirm or revise molecular structures, invent and test new synthetic methods, push chemists to develop creative strategies, train and equip the next generation synthetic chemists with the skills to tackle increasingly complex synthetic challenges,^7^ and impact the related disciplines especially biology and medicine.^8^ Total synthesis now aims not only to replicate nature’s complexity but often to surpass it.^9^

As the field of total synthesis evolves, various concepts and retrosynthetic logics have been formulated to guide synthetic design, push the boundaries, and make total synthesis esthetical, practical, and useful.^10−14^ Among them, molecular editing has emerged as an attractive concept. The term was first coined by Danishefsky in the context of diverted total synthesis to improve the biological function of the target natural products by editing unnecessary or even undesirable structural features.^15^ Recently, the concept of molecular editing was further broadened,^16^ and its impact went from total synthesis to drug discovery, material science, and other fields.^17^ Skeletal editing, as a converse of peripheral editing^18^ and functional group interchange, allows precise skeletal transformation or structural reorganization^19^ by means of insertion, deletion, atom/group exchange, rearrangement, and others. While plenty of classical synthetic transformations including Baeyer–Villiger oxidation, Beckmann rearrangement, Schmidt–Aubé reaction, Büchner–Curtius–Schlotterbeck reaction, Ramberg–Bäcklund ring contraction, Favorskii rearrangement, and Wolff rearrangement fall into the category of skeletal editing, the recent formulation of the skeletal editing concept especially single-atom skeletal editing has been fueling this field and inspiring many innovative and precise editing methods with potential for late-stage and complex structural editing.^20−26^

Skeletal editing intentionally or unintentionally has come to impact natural product total synthesis.^27,28^ For example, classic skeletal editing or topological rearrangement reactions including those mentioned earlier have been widely used at different stages in total synthesis. Classic examples include Corey’s erythronolide B synthesis,^29^ Woodward’s erythromycin A synthesis,^30^ Danishefsky’s compactin synthesis,^31^ Overman’s strychnine synthesis,^32^ Wood’s synthesis of staurosporine,^33^ Aubé’s stenine synthesis,^34^ Nicolaou’s hirsutellone B synthesis,^35^ and many others. Recent examples include the total syntheses of hippolachnin from Trauner,^36^ granatumine A from Newhouse,^37^ vinigrol from Li,^38^ piperarborenine B from Antonchick,^39^ harringtonolide from Sarpong,^40^ and many others. Due to the word limit and scope of this Account, details of many classic and contemporary examples are not discussed. However, this topic certainly merits comprehensive reviews. We focus on detailing five total synthesis examples from our research group using skeletal editing-based retrosynthetic logic. For each synthesis, we highlight the build–edit–decorate workflow and the magic editing moments that helped us navigate structural complexity and achieve our synthesis goals. We employ the spacial score (SPS) as a practical metric to track topological complexity and visualize scaffold evolution throughout each synthesis,^41^ which aligns with its recently established use by Sarpong to ensure consistency in examining complexity economy across synthetic studies.^12^ As revealed along the way, although SPS effectively captures structural growth, we acknowledge its limitations in recognizing the impact of the strategically important editing steps that in most cases barely move the SPS needle. While the edit steps appear “invisible” to the SPS topological metric, they are ultimately decisive in achieving the total synthesis.

## One-Carbon Heteroaromatic Skeletal Editing Enabled Total Synthesis of Complanadine A

**Figure 1:**
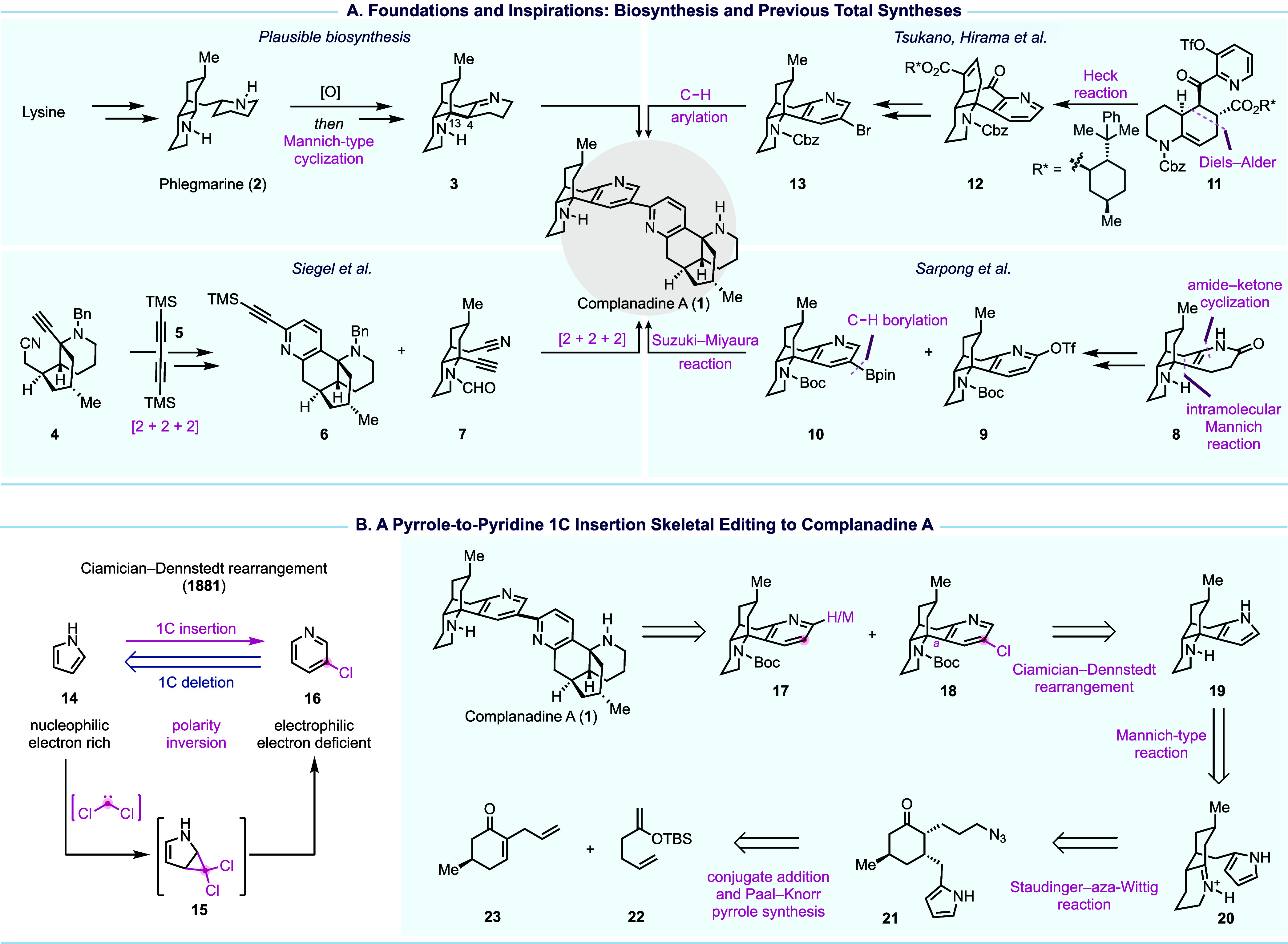
Plausible complanadine A biosynthesis, prior synthesis, and our strategy.

Complanadine A (**1**, Figure 1) belongs to the *Lycopodium* alkaloid family, one of the most skeletally diverse families of natural products isolated to date. Complanadine A is distinguished by its pyridine-containing polycyclic and heterodimeric skeleton and unique biological activity. It has demonstrated significant neurotrophic potential by enhancing nerve growth factor biosynthesis in human astrocytoma and glial cells, making it a promising candidate for neurological disorders. Inspired by its complex architecture, potent biological activity, and scarce natural abundance, we and others have undertaken its total synthesis to enable further biological evaluation.

Biosynthetically, the skeletal system of complanadine A is proposed to arise via an oxidation followed by a Mannich-type cyclization of phlegmarine (**2**), which itself can be derived from lysine (Figure 1A). Previous total syntheses of complanadine A have laid a strong foundation for exploring its complex architecture and biological relevance. In 2010, Siegel’s group reported an 18-step synthesis that features two remarkable Co-mediated [2 + 2 + 2] cyclizations to form the bipyridine moiety.^42^ Their synthesis enabled target identification, leading to the discovery of Mas-related G-protein-coupled receptor X2 as its potential cellular target for chronic pain management.^43^ Meanwhile, Sarpong and co-workers achieved a 15-step synthesis employing a biomimetic tandem 1,4-addition/Mannich cyclization/amide–ketone condensation to form key intermediate **8** and a combination of Ir-catalyzed C–H borylation and Suzuki–Miyaura cross coupling.^44^ In 2013, Tsukano and co-workers reported a 20-step synthesis featuring an intramolecular Heck reaction to build the tetracyclic core and a Pd-catalyzed C–H arylation to forge the C2–C3′ bipyridine linkage.^45^ These efforts collectively highlight the ingenuity required to address the synthetic challenges posed by complanadine A and set the stage for further innovations in its synthesis.

In 2021, we reported our total synthesis of complanadine A,^1^ showcasing the first use of Ciamician–Dennstedt rearrangement^46^ in total synthesis. Retrosynthetic disconnection of the bipyridyl linkage led to cross-coupling partners **17** and **18** with a tetracyclic skeleton (Figure 1B). For such a tetracyclic skeleton, retrosynthetic disconnection of bond **a** (see **18**) was considered strategic as it significantly reduced structural complexity. However, such disconnection would require a nucleophilic addition to an iminium ion which is difficult for an electron deficient pyridine group. On the other hand, it should be straightforward for an electron-rich pyrrole group known for its nucleophilicity. Thus, using skeletal-editing retrosynthetic logic, we traced **18** back to **19**. In the forward sense, this logic requires a one-carbon insertion strategy to convert the pyrrole precursor into the pyridine framework, ideally functionalized with a handle for the subsequent cross coupling. The Ciamician–Dennstedt rearrangement first discovered in 1881 was uniquely suited to this role, as it proceeds through a dihalocarbene cycloaddition on a pyrrole double bond, followed by ring expansion to yield a 3-chloropyridine (**14** → **16**). With this in mind, we further envisioned constructing **19** from **21** via a tandem sequence of Staudinger–aza-Wittig reaction and Mannich-type cyclization. Compound **21** was traced back to the simple building blocks **22** and **23**.

**Figure 2:**
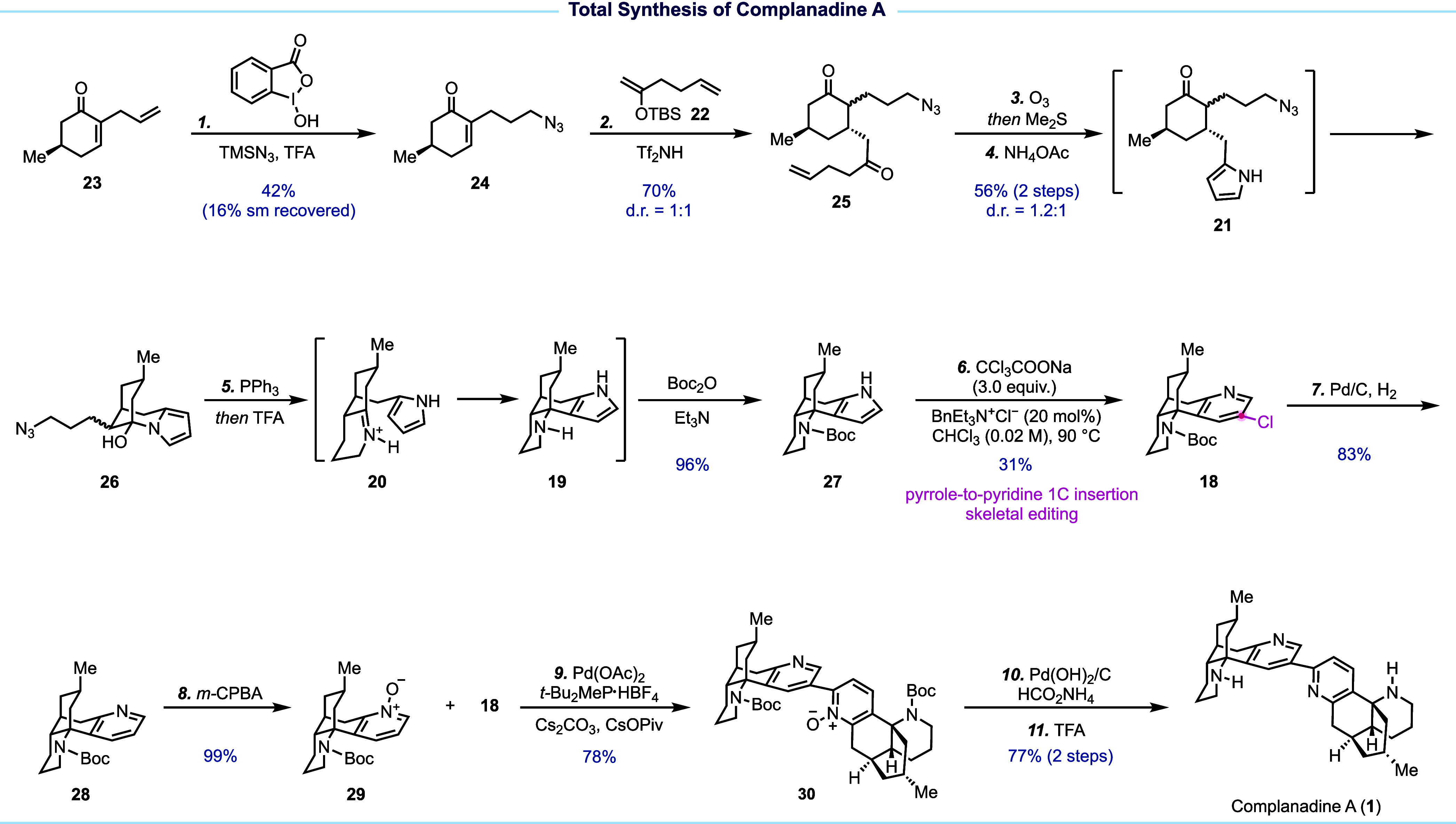
Total synthesis of complanadine A.

Our synthesis started from known compound **23** (Figure 2, prepared from chiral pool molecule (*R*)-(+)-pulegone in three steps or via an asymmetric organocatalysis in one step. An *anti*-Markovnikov hydroazidation converted **23** to **24** for the subsequent Mukaiyama conjugate addition, which resulted in a mixture of inconsequential diastereomers. This mixture was then subjected to ozonolysis and Paal–Knorr pyrrole synthesis to yield **21**, which spontaneously underwent cyclization to give **26**. From there, a one-pot sequence comprising Staudinger reduction, imine formation, Mannich-type cyclization, and Boc protection was employed to efficiently construct the tetracyclic framework, giving **27** in 96% yield. **27** was next transformed into 3-chloropyridine **18** via the Ciamician–Dennstedt one-carbon insertion. After removal of the chloride group, **28** was oxidized to **29** for subsequent palladium-catalyzed C–H arylation with **18** to furnish **30**. The latter was advanced to complanadine A to conclude an 11-step synthesis from **23**.

**Figure 3:**
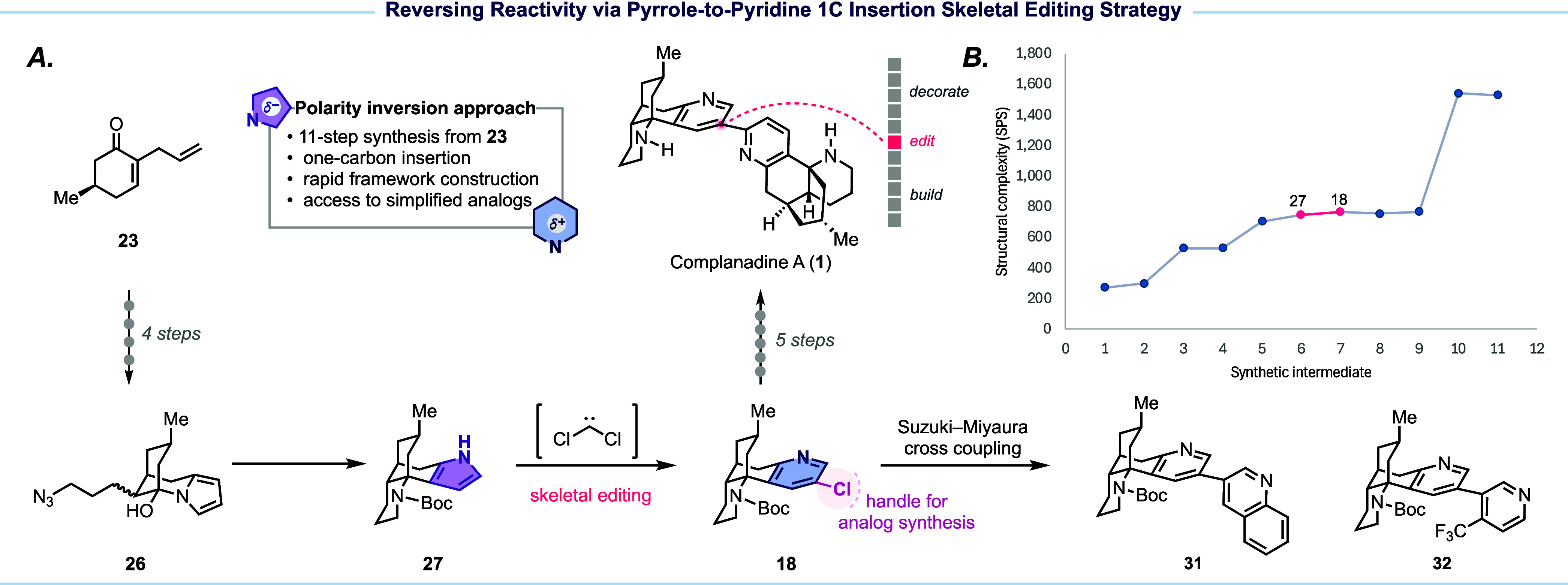
Summary and SPS analysis of our complanadine A synthesis.

In summary (Figure 3A), our synthesis starts with a five-step sequence to build key intermediate **27**, followed by Ciamician–Dennstedt rearrangement to precisely edit the pyrrole group to the desired 3-chloropyridine for subsequent C–H arylation and further decorations. While the pyrrole-to-pyridine one-carbon insertion editing step (**27** → **18**) appears as a relatively flat region on the SPS plot (Figure 3B), it allows us to leverage the nucleophilicity of pyrrole for rapid access to **27** while inverting of the aromatic system’s electronic properties and introducing a strategically positioned chlorine handle for subsequent cross-coupling to complete the total synthesis and create synthetic analogs (cf. **31**–**32**) for further biological evaluations.^47^ The SPS plot reflects macroscopic changes in structural complexity but often overlooks atomic-level transformations such as the one that occurs in the key skeletal editing step in this case. Additionally, the SPS plot shows only a marginal increase in complexity from **26** to **27** and does not capture the significant molecular reorganization. This transformation imposes new spatial constraints and topological features to significantly increase the molecule’s “encoded information content”, representing a critical yet understated gain in complexity that is not immediately visible on the SPS plot.

## One-Carbon Carbocycle Skeletal Editing Enabled Total Syntheses of Phleghenrines A and C

**Figure 4:**
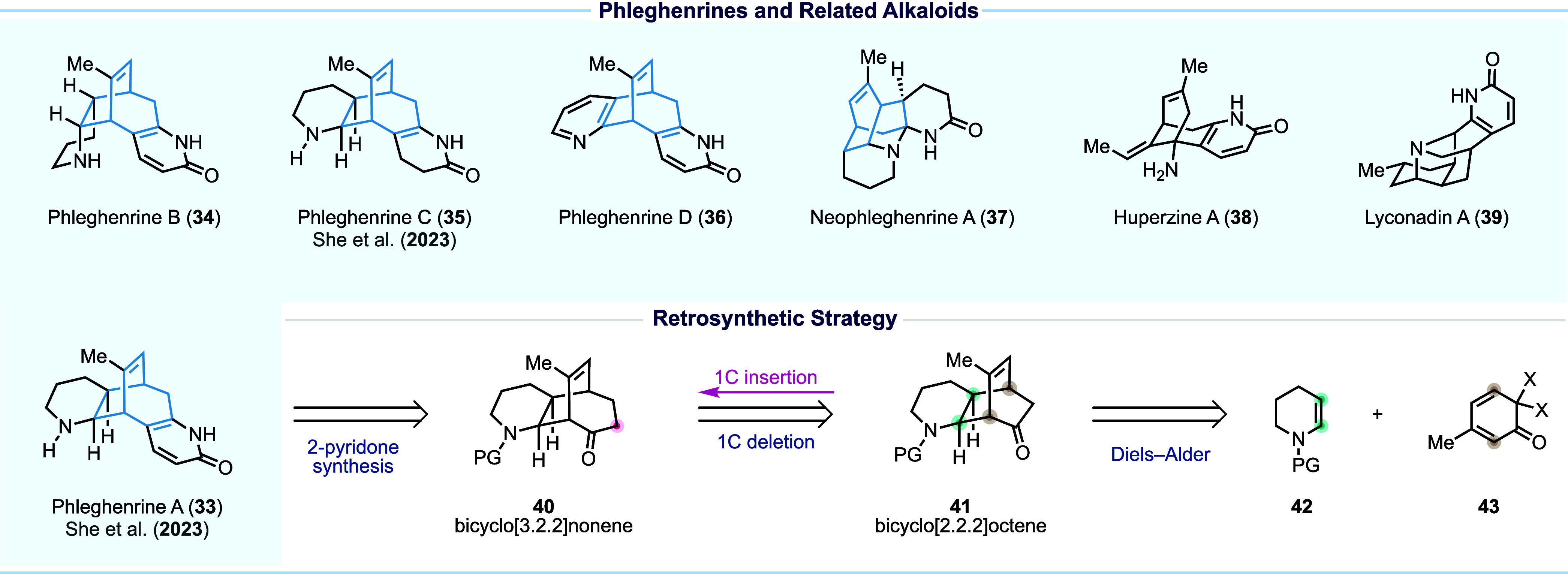
Phleghenrine alkaloids and our retrosynthetic analysis.

The phleghenrines (**33**–**37**, Figure 4) were isolated from *Phlegmariurus henryi* Ching, also known to produce *Lycopodium* alkaloids such as huperzine A (**38**). Similar to huperzine A (**38**), the phleghenrines exhibit acetylcholinesterase inhibition, with phleghenrines A and D identified as the most active family members but with low or no inhibition activity against the butyrylcholinesterase, indicating their potential as valuable leads for neurodegenerative disease treatment. The phleghenrines have a unique and complex tetracyclic skeleton featuring a bicyclo[3.2.2]nonene core and a 2-pyridone or its derivative. Their therapeutic potential, intriguing topological structure, and low isolation yield (0.0003%) make them attractive molecules for total synthesis. In 2023, She and co-workers reported their total syntheses of phleghenrines A and C in 23 and 22 steps, respectively.^48^

Our long-standing interest in *Lycopodium* alkaloids, such as complanadine A (**1**) and lyconadin A (**39**),^49^ led us to the phleghenrines. One challenge associated with phleghenrine synthesis lies in their bicyclo[3.2.2]nonene core, which is fused with a piperidine/pyridine on one side and a 2-pyridone or its derivative on the other side. Accessing such a framework presents a difficult task due to its inherent complexity and the lack of efficient methods for its construction. In contrast, the bicyclo[2.2.2]octene system is far more accessible and invites several Diels–Alder disconnections. Thus, we decided to use a bicyclo[2.2.2]octene as the precursor of the desired bicyclo[3.2.2]nonene framework by leveraging a one-carbon insertion skeletal editing strategy, which would bypass the difficulties associated with constructing the bicyclo[3.2.2]nonene motif directly. Retrosynthetically, we planned to install the 2-pyridone moiety in the decorate stage on intermediate **40** and proposed bicyclo[2.2.2]octenone **41** as the precursor to **40**. This retrosynthetic design allowed us to construct the bicyclo[2.2.2]octenone core early in the sequence via an inverse electron-demand Diels–Alder reaction between *N*-protected enamine **42** and masked *o*-benzoquinone (MOB) **43**. Since both dienophiles of type **42** and dienes of type **43** tend to undergo homodimerization reactions, the success of this strategy hinges on identifying a pair of proper diene and dienophile for the Diels–Alder reaction and a strategic one-carbon insertion with precision to expand the bicyclo[2.2.2]octenone system into the bicyclo[3.2.2]nonenone framework. For the latter, we planned to take advantage of the ketone reactivity by employing the Büchner–Curtius–Schlotterbeck reaction to enable the desired ring expansion.^50^

**Figure 5:**
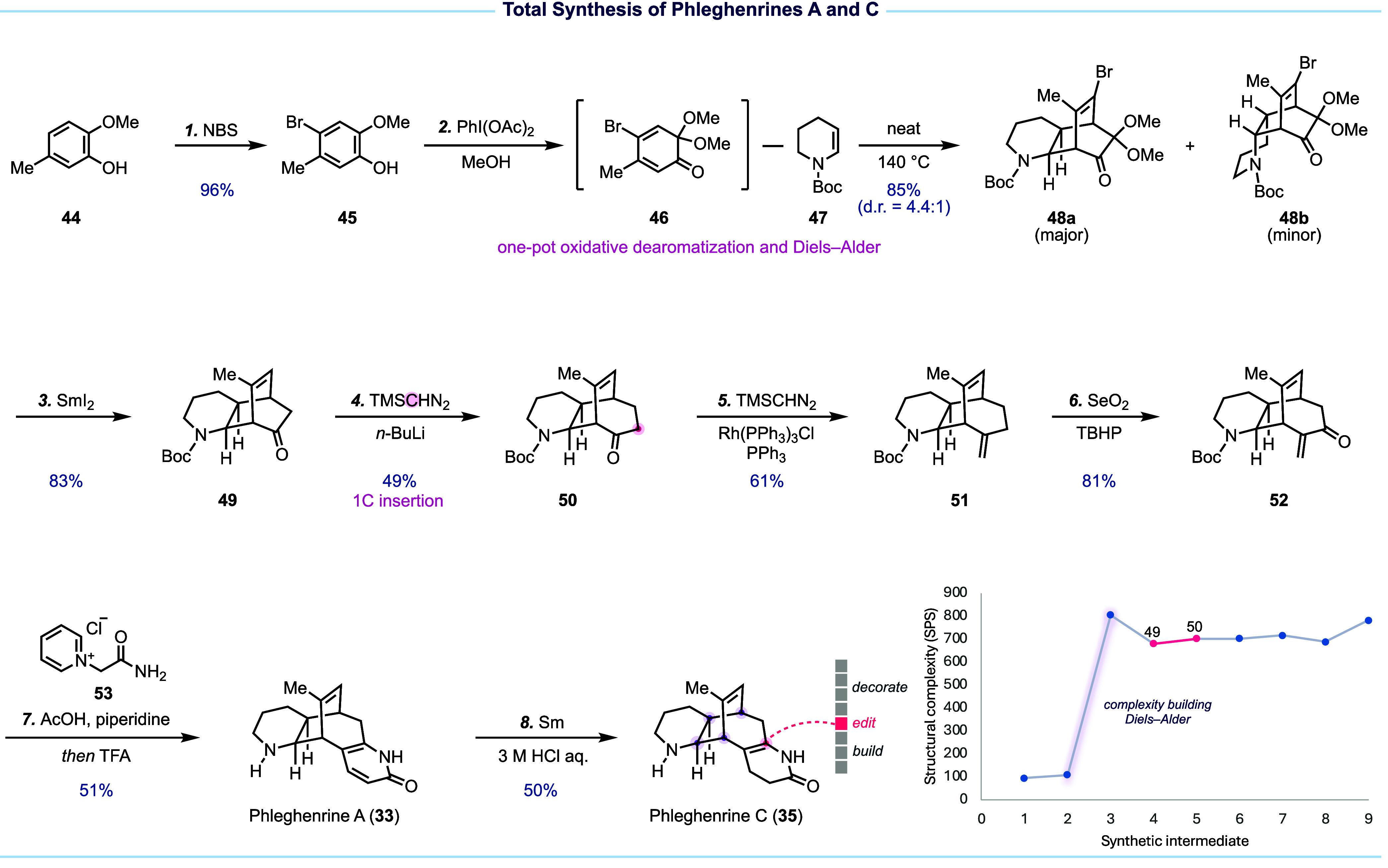
Total synthesis of phleghenrines A and C and the related SPS analysis.

Our synthesis (Figure 5) starts with building the bicyclo[2.2.2]octenone intermediate via the proposed inverse electron-demand Diels–Alder reaction, which proved to be nontrivial. Eventually, MOB **46** with an extra bromine atom was identified as the proper diene. The bromide substitution is important to prevent self-Diels–Alder dimerization. *N*-Boc protected enamine **47** was proved to have matched reactivity with **46**. Diene **46**, prepared from commercially available **44** via bromination and oxidative dearomatization, was trapped in situ with **47** to deliver a separable 4.4:1 mixture of **48a** (major) and **48b** in 85% yield. Notably, this early complexity-building event as reflected in the SPS plot, achieving a remarkable +696 SPS increase (650% boost), rapidly sets the requisite bicyclo[2.2.2]octenone core and provides a platform for subsequent functionalization. The extra bromide and acetal group of **48a** was reductively removed with SmI_2_, providing tricyclic intermediate **49** for the editing step with the Büchner–Curtius–Schlotterbeck one-carbon insertion, which successfully expanded the ring system of **49** to the desired bicyclo[3.2.2]nonenone **50**. Again, the strategic importance of this one-carbon insertion was not captured by the SPS calculation because only one methylene group is added, while the system evolves topologically rather than dramatically growing in size. With **50** in hand, we entered the decorate stage. Wittig one-carbon homologation using the Lebel modification delivered **51** for subsequent allylic C–H oxidation to provide α-methylene ketone **52**. Finally, a one-pot 2-pyridone synthesis and Boc-deprotection completed phleghenrine A and partial reduction of phleghenrine A with Sm led to phleghenrine C. Overall, this synthesis achieves high efficiency by combining rapid framework construction, precise skeletal editing, and minimum late-stage decorations to reach phleghenrines A and C in just 7 and 8 steps, respectively.^2^

## Cut-and-Insert Skeletal Editing Enabled Total Synthesis of Crinipellins A and B

**Figure 6:**
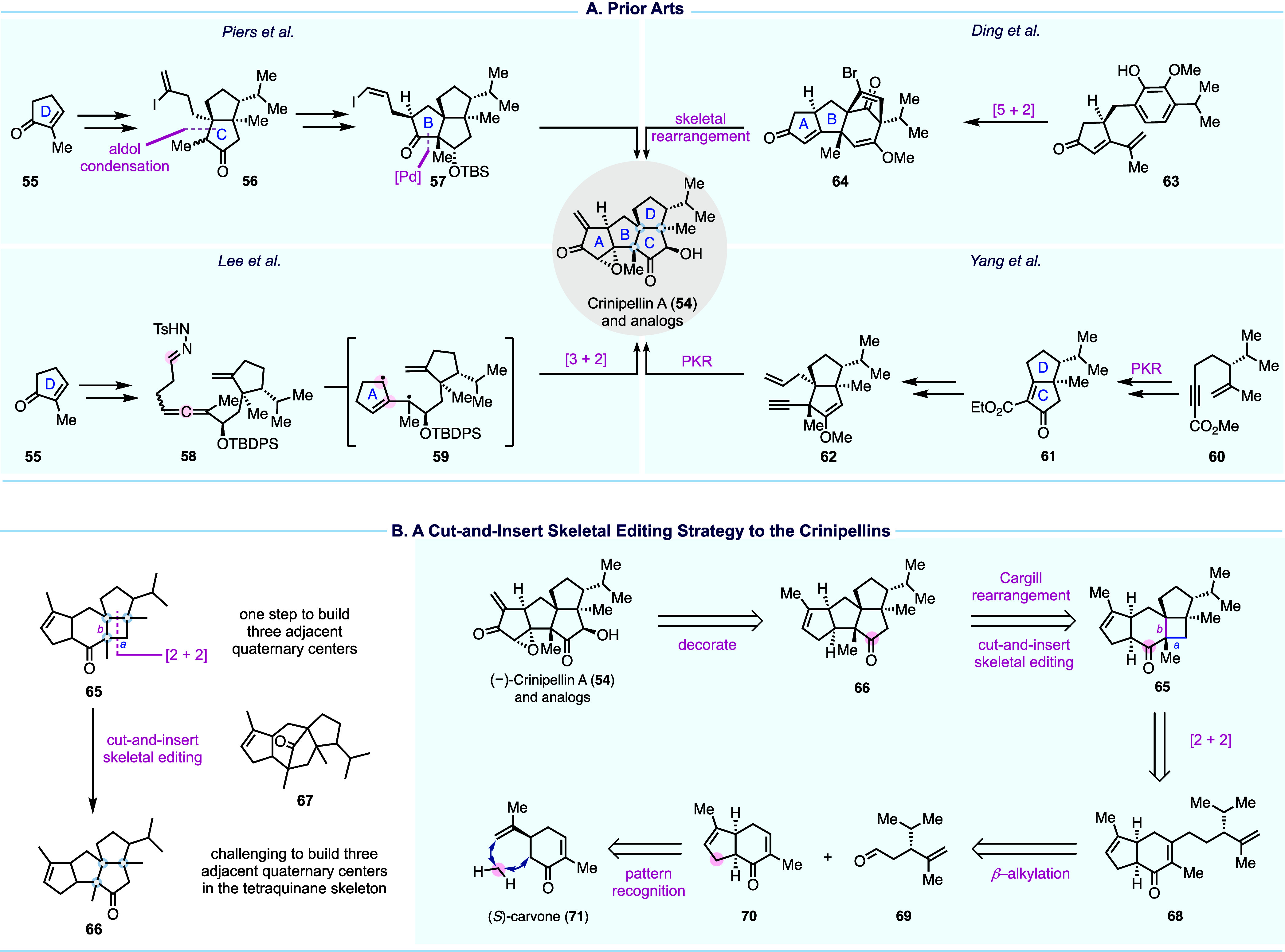
Prior crinipellin total syntheses and our strategy.

Crinipellins A and B (Figure 6A) represent a rare class of natural products with a tetraquinane core. Their highly congested tetracyclic ring system, eight contiguous stereocenters, including three all-carbon quaternary centers, and multiple oxygenated functionalities make them challenging synthetic targets. This complexity, coupled with their isolation burden, broad-spectrum bioactivity, and elusive biological targets, has rendered crinipellins attractive target molecules for total synthesis. Beyond their structural allure, the rare combination of the α-methylene ketone and α,β-epoxide moieties allows for exploration of bivalent reactivity with nucleophilic sites, e.g., cysteines, for yet-to-be-identified cellular proteins.

In 1993, Piers and Renaud achieved the first total synthesis of (±)-crinipellin B in 22 steps. Their approach elegantly employed a series of carbonyl transformations to build the ABC ring system.^51^ In 2014, Lee and co-workers disclosed a 32-step total synthesis of (−)-crinipellin A featuring a remarkable tandem sequence of [3 + 2] cycloaddition, nitrogen extrusion, and radical cyclization to build the BC ring system.^52^ In 2018, Yang and co-workers reported their total syntheses of (−)-crinipellins A and B in 17 and 18 steps, respectively. Their synthesis utilizes two Pauson–Khand reactions (PKR) to build the CD and AB ring systems.^53^ In 2022, Ding and co-workers developed a divergent approach to access seven crinipellin congeners (14–18 steps) including crinipellins A and B in 16 steps each.^54^ Their synthesis features an oxidative dearomatization-induced [5 + 2] cycloaddition to access **64**, which was later rearranged to the crinipellin carbon skeleton via a hydrogen atom transfer initiated structural rearrangement.

**Figure 7:**
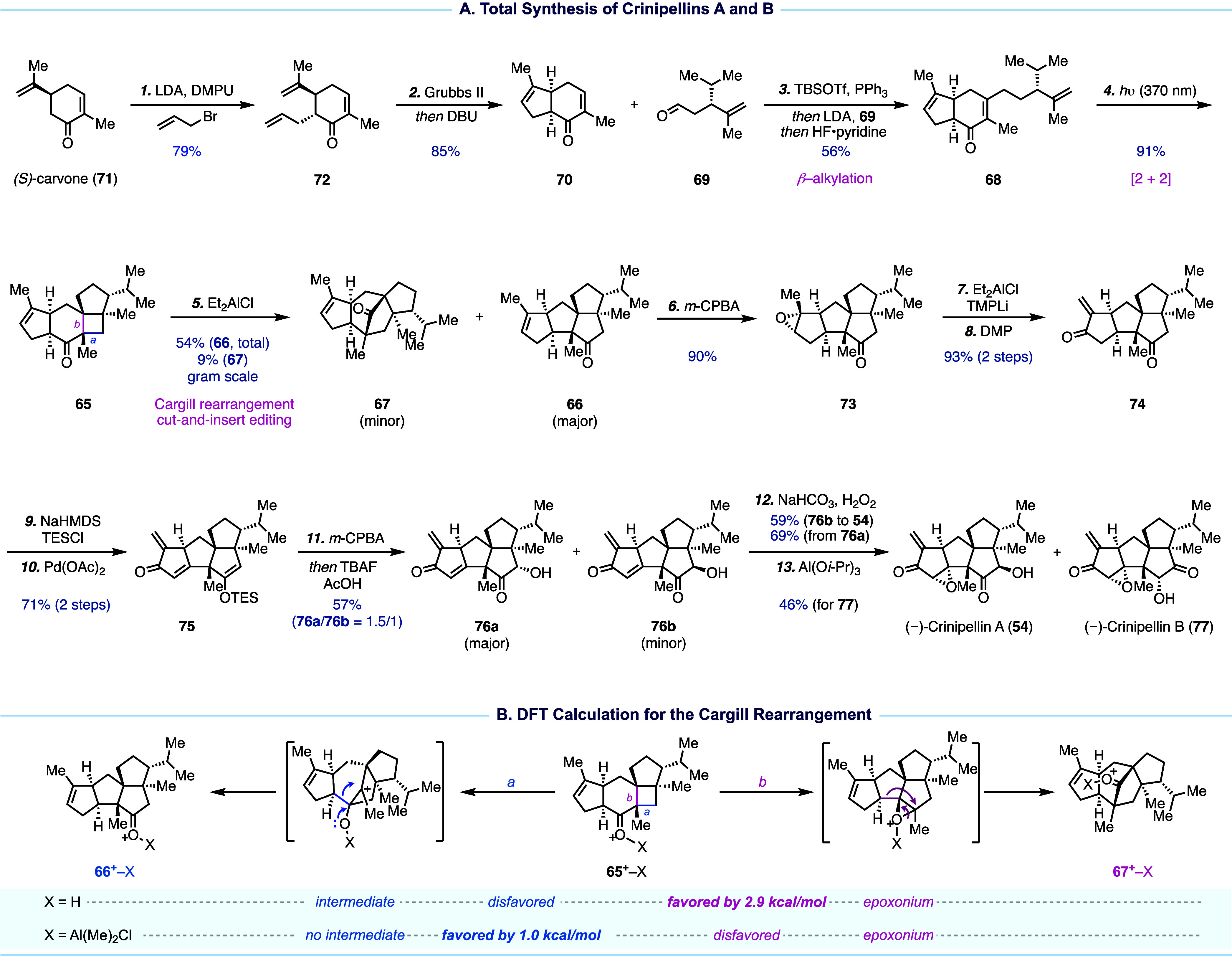
Total synthesis of crinipellins and DFT calculation.

Total synthesis of the crinipellins is challenged by its highly congested and decorated tetraquinane core. As evidenced by previous syntheses, directly building the tetraquinane system with three contiguous all-carbon quaternary centers can be tedious and requires powerful synthetic transformations. Rearranging a more accessible skeleton to the tetraquinane skeleton, as seen in Ding’s approach, can bypass this direct challenge but would need a strong driving force to promote the desired structural rearrangement and an efficient strategy to prepare the precursor. Based on this logic, we proposed to access tetraquinane skeleton **66** from **65** with a 5/6/4/5 tetracyclic system. In the forward sense, a cut-and-insert skeletal editing process is required to cut out the carbonyl group in the cyclohexanone and insert it into the cyclobutane ring of **65**. To realize such a skeletal editing process, we resorted to the Cargill rearrangement.^55^ Strain release was considered the driving force for the proposed Cargill rearrangement, but this rearrangement could proceed in two different directions depending on whether bond **a** or **b** migrates first, leading to desired product **66** or undesired product **67**, respectively (also see Figure 7B). Thus, controlling the Cargill rearrangement with precision was needed. More importantly, unlike tetraquinane **66**, tetracyclic intermediate **65** could be accessed via a photochemical [2 + 2] cycloaddition, a reliable method for building congested ring systems and all-carbon quaternary centers. This led us to **68** as the [2 + 2] cycloaddition precursor, which could be accessed via a formal β-alkylation of **70** with aldehyde **69**, utilizing the method developed by Kozikowski.^56^ Pattern recognition analysis^57^ traced **70** back to chiral pool molecule (*S*)-carvone (**71**). (*S*)-Carvone (**71**) is only one carbon atom away from **70**, but such a one-carbon insertion, though appealing, does not yet exist, thus requiring a detour.

Our synthesis begins with converting (*S*)-carvone to bicyclic intermediate **70** via a two-step detour: α-allylation and ring-closing metathesis with one-pot epimerization of the α-stereocenter. Kozikowski formal β-alkylation of **70** with **69** delivered **68** for the [2 + 2] cycloaddition, which efficiently generated tetracyclic **65** in 91% yield. The subsequent Cargill rearrangement required comprehensive optimization. Eventually, Et_2_AlCl was identified as an optimal promoter, delivering the desired tetraquinane **66** as the major product, alongside minor formation of the undesired product **67**. Notably, when *p*-TsOH, Tf_2_NH, ZnBr_2_, InCl_3_, AlCl_3_, or BF_3_·Et_2_O was used for the Cargill rearrangement, **67** was produced as the dominant product, highlighting the sensitivity of the rearrangement toward the promoting reagents. To further understand the rearrangement mechanism, DFT calculations (SMD(toluene)-mPW1PW91/6-31+G(d,p)) were conducted (Figure 7B) for the systems involving **65**^**+**^-H and **65**^**+**^-Al(Me)_2_Cl. For **65**^**+**^-H, the formation of both **66**^**+**^-H and **67**^**+**^-H was discovered to involve stepwise dyotropic rearrangements, favoring the formation of **67**^**+**^-H by 2.9 kcal/mol. For **65**^**+**^-Al(Me)_2_Cl, concerted dyotropic reactions (albeit involving asynchronous alkyl shifting events) were suggested and the formation of **66**^**+**^-Al(Me)_2_Cl was favored by only 1.0 kcal/mol. With **66** in hand, the remaining objective was to decorate the tetraquinane skeleton by introducing additional oxygen functionality and adjusting the oxidation state through a series of carefully planned redox manipulations. Selective epoxidation of **66** delivered **73**. Subsequent epoxide ring opening and DMP oxidation produced α-methylene ketone **74** for the following selective Saegusa–Ito oxidation to deliver **75** with a spared TES enol ether which underwent Rubottom oxidation to introduce the α-hydroxy ketone moiety in the C ring, yielding a 1.5/1 mixture of **76a** and **76b**. Selective nucleophilic epoxidation of the more strained enone in the A ring of **76b** completed a 12-step total synthesis of (−)-crinipellin A. For (−)-crinipellin B, after nucleophilic epoxidation of **76a**, an additional step was used to isomerize the α-hydroxy ketone in the C ring to complete a 13-step synthesis.

**Figure 8:**
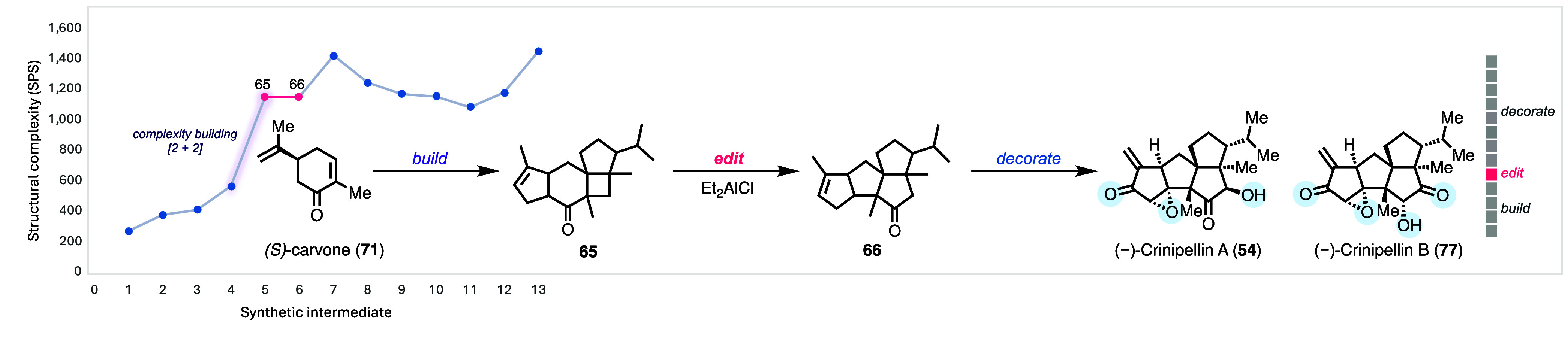
Summary and SPS analysis of our crinipellin total synthesis.

In summary (Figure 8),^3^ the success of our crinipellin total synthesis relies on a rapid escalation in molecular complexity during the early stages of the synthesis.^12^ The skeletal-editing retrosynthetic logic revealed **65** as the precursor of tetraquinane **66**. Identification of this latent structural relationship allows us to use the six-membered ring containing (*S*)-carvone as the starting material and leverage the power of photochemical [2 + 2] cycloaddition to build a strained cyclobutane ring embedded with three contiguous all-carbon quaternary centers. The latter rapidly generates complexity, achieving a dramatic +880 SPS increase (323% boost) from its precursor. The energy built in the cyclobutane ring then drives the Cargill rearrangement under precisely controlled conditions to provide **66** for the subsequent decorations en route to crinipellins A and B. Although the editing step does not increase the SPS complexity, it remains the most strategically important maneuver in efficiently realizing our target.

## Skeletal Reorganization and Rearrangement Enabled Efficient Synthesis of GA_18_ Methyl Ester

**Figure 9:**
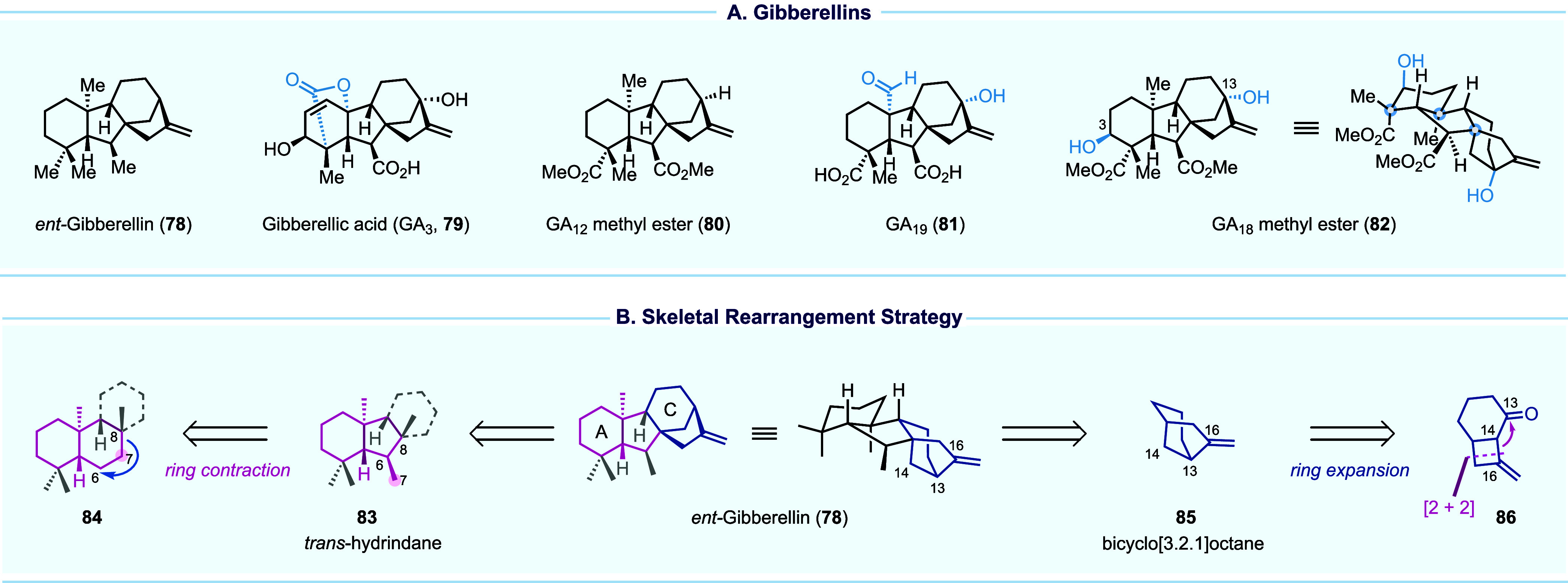
Gibberellins and our synthetic strategy.

The gibberellins (GAs) have been widely used in modern agriculture as plant hormones to regulate a variety of plant developmental processes. Representative GA members include *ent*-gibberellin (**78**, Figure 9A, the parent compound), GA_3_ (**79**), GA_12_ (**80**), GA_19_ (**81**), and GA_18_ (**82**). The biological significance, coupled with the structural complexity and diversity of gibberellins, has rendered them attractive targets for synthetic chemists. The first landmark total synthesis of gibberellic acid (GA_3_) was achieved by Corey and co-workers in 1978, followed by elegant syntheses from Mander and others.^58−61^

We reported our synthesis of GA_18_ methyl ester in 2023.^4^ GA_18_, a C20 gibberellin family member, was isolated from the immature seeds of *Lupinus luteus* in an extremely low yield (0.000058%). Limited studies showed that in d_1_ and d_5_ mutants of maize, GA_18_ is as active as GA_3_. Structurally, GA_18_ features the *ent*-gibberellin tetracyclic skeleton with eight chiral centers including three all-carbon quaternary centers and a unique hydroxylation pattern at C3 and C13. In general, the challenge of gibberellin synthesis lies in how to construct their tetracyclic carbon skeleton and precisely decorate the carbon skeleton with appropriate oxygen functionalities. In our structural analysis (Figure 9B), we dissected the tetracyclic carbon skeleton into two sections: one featuring a *trans*-hydrindane (**84**) and the other one containing a bicyclo[3.2.1]octane (**85**), both of which are challenging structural motifs. Inspired by their biosynthetic pathways, we envisioned a *trans*-decalin (cf. **84**) as the precursor of *trans*-hydrindane (**83**). The former is relatively accessible and can often be found in abundant natural products, but a suitable ring contraction method was necessary to convert **84** to **83**. For the bicyclo[3.2.1]octane ring system (**85**), we envisioned a 6/4-fused ring system (**86**) as its precursor. While structures like **86** could be quickly assembled with an allene–enone [2 + 2] cycloaddition, a precise skeletal editing method was required to migrate C16 from C14 to C13 and realize a ring expansion.

**Figure 10:**
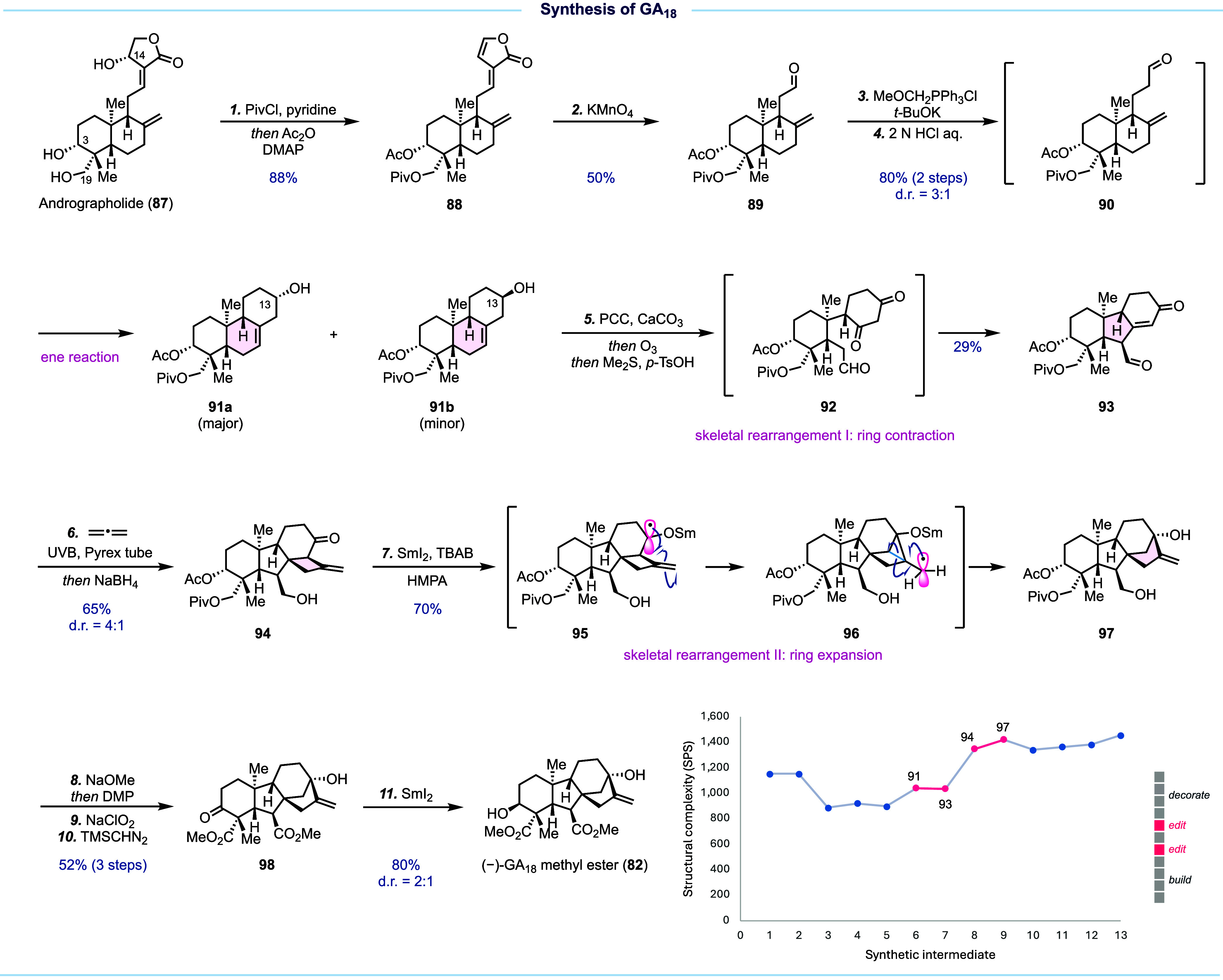
Synthesis of GA_18_ and SPS analysis.

Guided by a skeletal rearrangement-driven approach and informed by pattern recognition analysis, we identified a cheap and abundant diterpenoid, andrographolide (**87**, $2/g), as the starting material for the synthesis of (−)-GA_18_ methyl ester (Figure 10). In addition to the *trans*-decalin ring system, andrographolide is equipped with the desired oxygenation pattern in the A ring and synthetic handles to construct the C ring. Our synthesis starts from degrading andrographolide to **90** for the subsequent intramolecular ene reaction to form the C ring. Andrographolide was first converted to **88** via global hydroxyl group protection and spontaneous C14 allylic acetate elimination. The next selective oxidative cleavage of the trisubstituted double bond was achieved with KMnO_4_ to deliver aldehyde **89** followed by a two-step one-carbon homologation to give **90**, which underwent intramolecular ene reaction under the hydrolysis step to give **91a** (major) and **91b** (minor) as a mixture of diastereomers. This mixture was next oxidized to the same ketone, setting the stage for the first skeletal reorganization via one-pot ozonolysis (to ketoaldehyde **92**) and intramolecular aldol reaction. This process contracted the *trans*-decalin to the *trans*-hydrindane encoded in the gibberellins. We then used an allene–enone photochemical [2 + 2] cycloaddition followed by aldehyde reduction to furnish **94** for the next skeletal rearrangement to convert the 6/4-fused bicycle to a desired [3.2.1] bridged bicycle. This skeletal editing step was achieved by using the method developed by Takatori and co-workers,^62^ which proceeded with a one-electron reduction of the C13 ketone to generate radical anion **95** for the next 3-*exo*-trig radical cyclization followed by subsequent selective C–C bond scission to deliver **97** in 70% yield. **97** contains the complete gibberellin carbon framework and the requisite oxygenation pattern of the target. The subsequent four decoration steps adjusted the oxidation state, fixed the stereochemistry at C3, and led to an 11-step synthesis of (−)-GA_18_ methyl ester.

In summary, a skeletal rearrangement-driven approach facilitated an efficient synthesis of (−)-GA_18_ methyl ester from andrographolide. Given the inherent complexity of andrographolide, SPS analysis showed a decrease in complexity in the first few steps. The first skeletal reorganization (**91** → **93**), while strategically important to build the *trans*-hydrindane, barely changes the SPS score. The rapid increase in structural complexity occurs during the allene–enone [2 + 2] cycloaddition step (**93** → **94**). The following skeletal rearrangement (**94** → **97**) is recorded as a modest increase on the SPS plot but is mechanistically profound and strategically important.

## Skeletal Rearrangement for Divergent Total Synthesis of Monoterpene Indole Alkaloids

**Figure 11:**
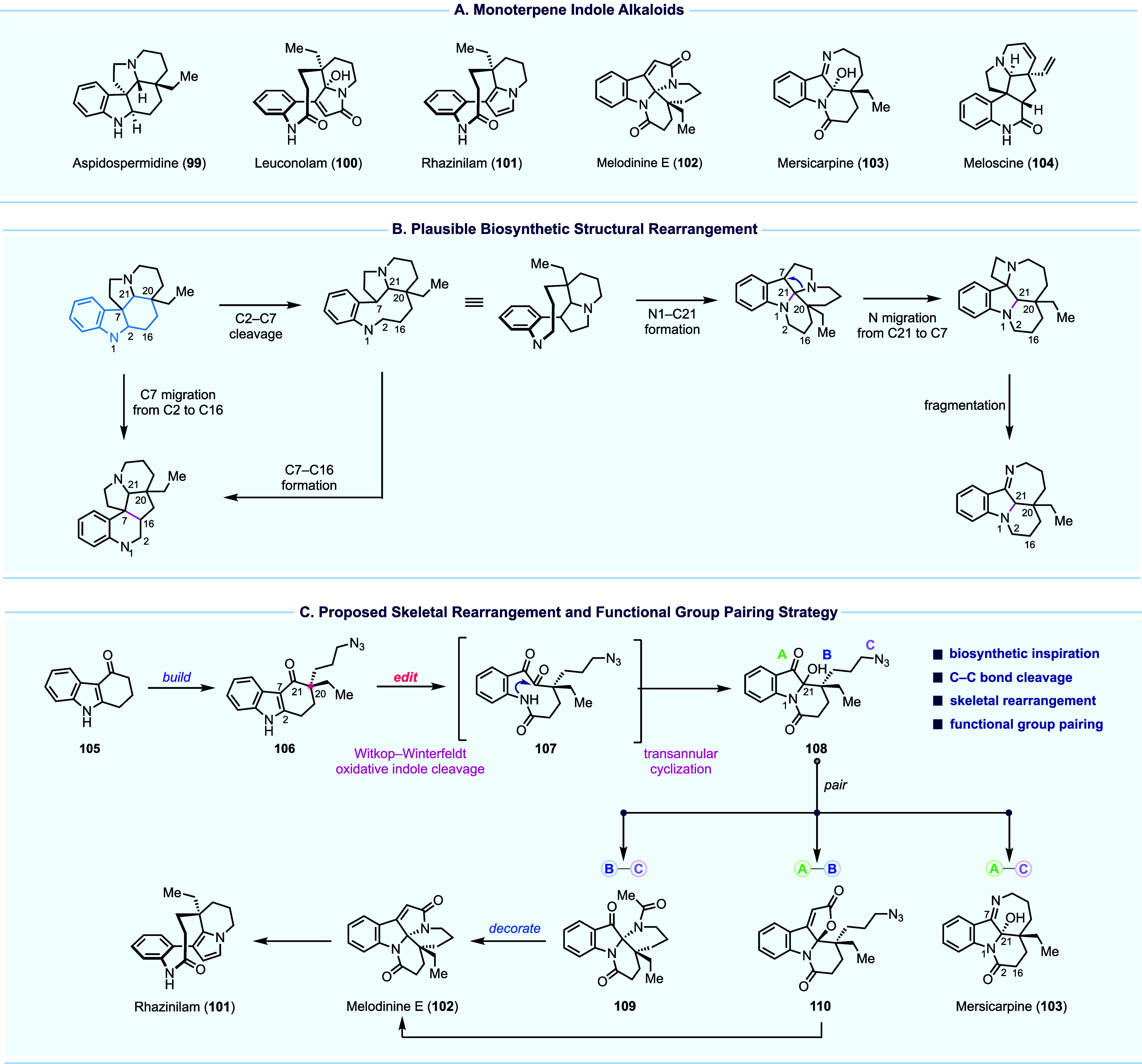
Monoterpene indole alkaloids, biosynthesis, and our divergent synthetic plan.

Terpene indole alkaloids have long stood out as one of the most influential families of natural products, responsible for lifesaving drugs such as vinblastine, vincristine, yohimbine, ajmalicine, ajmaline, quinine, and camptothecin. Since their discovery, these compounds have been at the forefront of synthetic efforts, reflecting their importance in both medicine and chemistry. Among terpene indole alkaloids, a collection of structurally rearranged monoterpene indole alkaloids (Figure 11A) such as leuconolam (**100**), rhazinilam (**101**), melodinine E (**102**), mersicarpine (**103**), and meloscine (**104**) have been isolated and reported with a broad range of biological activity. Biosynthetically, these structurally rearranged monoterpene indoles are proposed to be derived from the *Aspidosperma* subfamily such as aspidospermidine (**99**) via a series of C–C or C–N bond disconnection and recombination events (Figure 11B). These natural products have garnered a significant amount of synthetic attention, which resulted in numerous elegant total syntheses.^63−65^

Inspired by nature’s divergent biosynthetic pathways, at the beginning of our research program, we set out to develop a divergent approach^66^ focusing on accessing multiple targets through a combination of skeletal editing and functional group pairing^67^ (Figure 11C). Toward this goal, a readily accessible key intermediate with unique reactivity and functional groups that can engage in versatile functional pairing transformations was needed. Tricyclic compound **108** equipped with a ketone, a hemiaminal, and an azide was designed as this key intermediate. Selectively pairing its functional groups was expected to lead to mersicarpine (**103**), **109**, and **110**. From the latter two, melodinine E (**102**), rhazinilam (**101**), and other monoterpene indole alkaloids could be reached. To access **108**, we designed a Witkop–Winterfeldt oxidative indole cleavage^68,69^ of **106** followed by transannular cyclization to achieve the desired skeletal reorganization. Compound **106** could be derived from commercially available **105** by installing two alkyl substituents at the α-position of the ketone.

**Figure 12:**
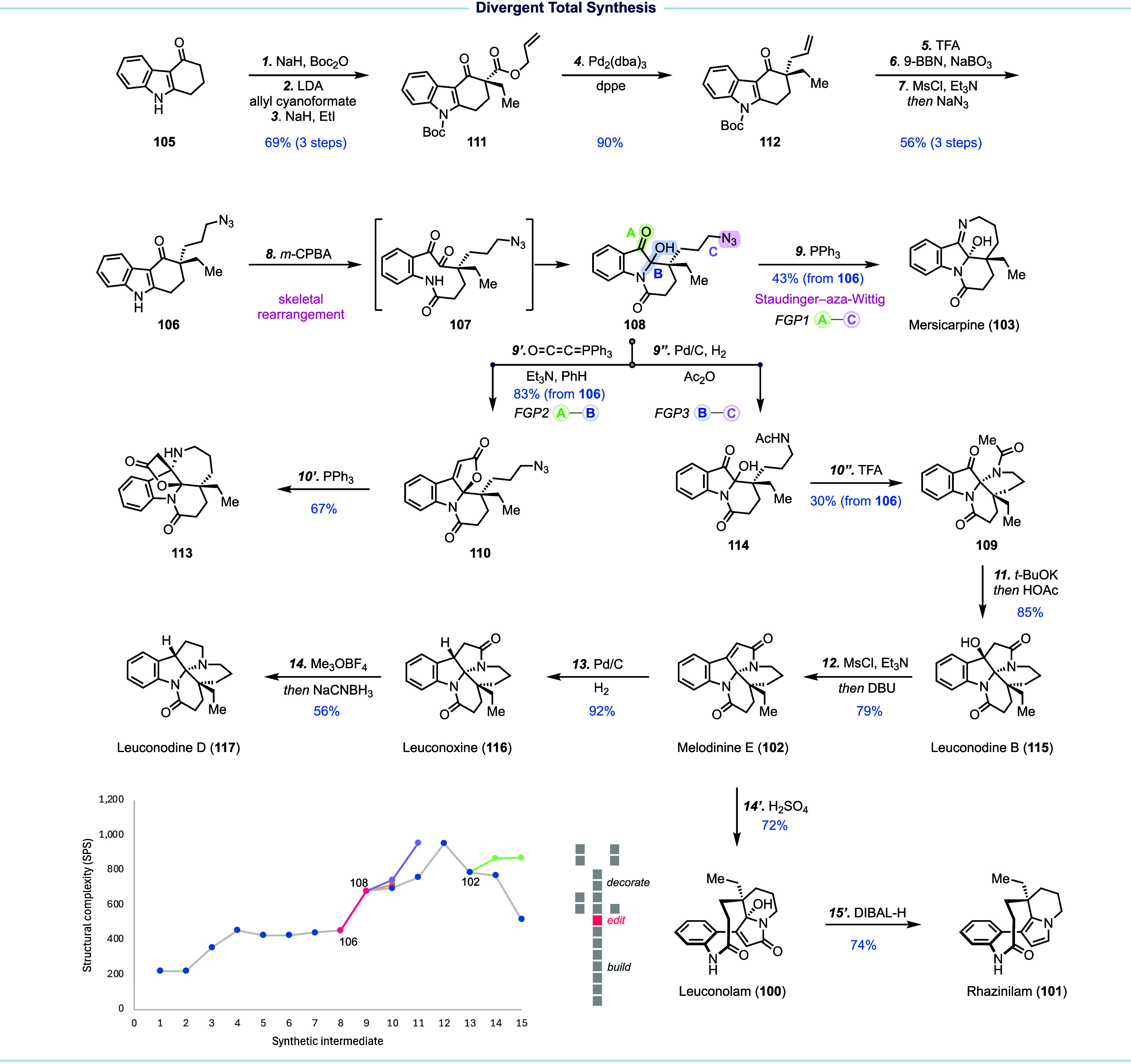
Divergent total synthesis of seven monoterpene indole alkaloids.

In the forward sense (Figure 12), **105** was advanced to **106** in seven synthetic transformations, including a palladium-catalyzed decarboxylative allylation to build the key all-carbon quaternary center. Oxidative cleavage of the C2–C7 double bond of the aromatic indole with *m*-CPBA afforded lactam **107**. This high-energy intermediate collapsed via spontaneous transannular cyclization to provide hemiaminal **108** as a 2:1 mixture of diastereomers. This rearrangement exhibits a +225 SPS score increase and effectively positions the synthesis at a node from which divergent routes can emanate. Exposure of **108** to Staudinger–aza-Wittig conditions delivered mersicarpine (**103**). The same intermediate **108** could also undergo Bestmann ketene lactonization to produce lactone **110**. Staudinger reduction of **110** with the original goal of accessing melodinine (**102**) revealed a previously unknown chemical entity (**113**) via a logic aza-Michael addition. While this result deviates from our total synthesis plan, it highlights the ability of divergent strategies to access a new chemical space. Concurrently, azide **108** was converted to acetamide **114** via catalytic hydrogenation and in situ amide formation. Upon acid treatment, cyclization occurred to generate Zhu’s intermediate^70^**109** for their leuconolam–leuconoxine terpene indole alkaloid syntheses. Built upon Zhu’s prior work, total syntheses of leuconodine B (**115**), melodinine E (**102**), and leuconoxine (**116**) were achieved. In addition, a chemoselective reduction of the more electron-rich γ-lactam in the presence of the more electron-deficient δ-lactam using Borch’s protocol^71^ converted leuconoxine (**116**) to leuconodine D (**117**), marking its first total synthesis. In parallel, melodinine E (**102**) was subjected to an acid-induced rearrangement to afford leuconolam (**100**). We next developed the first direct transformation of leuconolam to rhazinilam (**101**) via DIBAL-H reduction.

In summary, our divergent strategy was designed and executed with the ability to traverse a complex chemical space via orthogonal functional group pairing reactions of shared intermediate **108**. The skeletal rearrangement from **106** to **108** is key to the success of this synthetic campaign, resulting in the total syntheses of seven monoterpene indole alkaloids.

## Concluding Remarks

**Figure 13:**
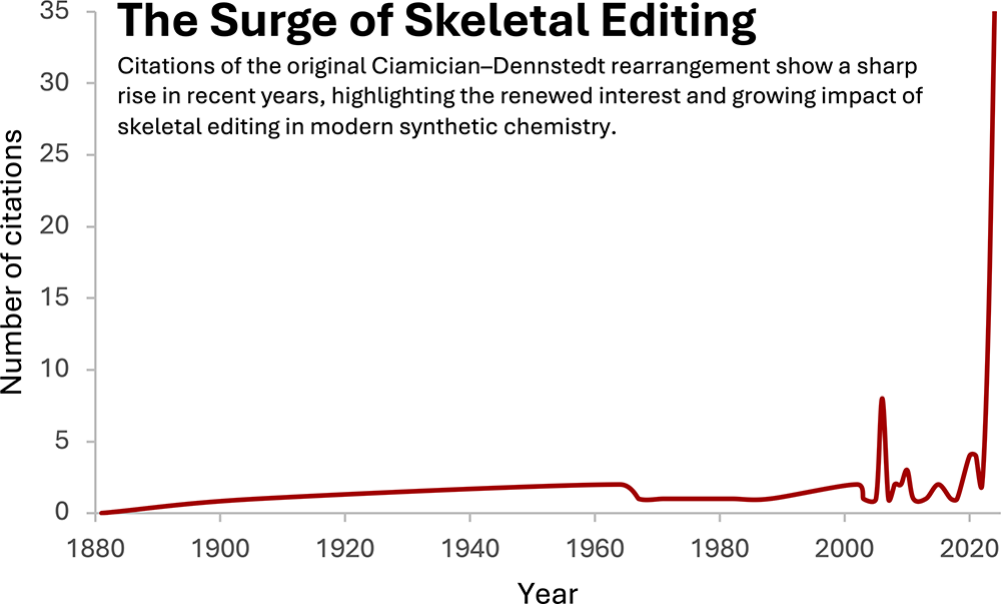
Surge of skeletal editing (citation numbers collected on December 9, 2024).

This Account highlights five of our total syntheses where skeletal editing played an essential role in achieving both a high standard of efficiency and aesthetic elegance. Through precise one-carbon insertions and controlled skeletal rearrangements, we completed the chemical syntheses of complanadine A, phleghenrines A and C, crinipellins A and B, GA_18_, and seven monoterpene indole alkaloids. Admittedly, at the beginning of our research program, skeletal editing was only sporadically employed based on our synthetic needs. However, as our total synthesis program and the skeletal editing field have evolved, this strategy has become an intentional and systematic practice in our synthesis design and execution. The citation trend of the original Ciamician–Dennstedt rearrangement paper reflects how the skeletal editing field has been developing (Figure 13). It was first reported in 1881 as a method way ahead of its time but was significantly overlooked for over 100 years.^46,72^ For most of the 20th century, this method remained underutilized, as reflected by minimal citations and its niche status in organic synthesis. A noticeable increase in interest was observed in the early 2000s, but the last four years have seen a dramatic spike in citations of the original paper. As a century-old discovery is revisited and adapted to meet modern synthetic challenges, we see how skeletal editing uniquely addresses the growing demand for methods that manipulate molecular frameworks postassembly with precision and equips chemists with tools to invent entirely novel molecular entities.

Skeletal editing-based retrosynthetic logic^73^ coupled with enabling skeletal editing methods are expected to inspire more creative synthetic strategies and improve synthetic efficiency and throughput. Skeletal editing can expand the scope of starting materials toward target molecules. For example, the benzene-to-pyridine skeletal editing^74,75^ could in principle allow the use of benzene as a pyridine precursor to eliminate issues with pyridine in terms of selectivity, compatibility, purification, etc. The pyridine group could be revealed at a later stage when the skeleton assembly and decoration are complete. The recently developed nitrogen deletion chemistry^20^ can allow the use of *N*-heterocycles as a starting point for carbocycles and leverage the various reactivities of *N*-heterocycles that do not exist for carbocycles.^39^ Skeletal editing strategies can also change or even reverse the reactivity and polarity to realize transformations that would otherwise be difficult or impossible. By toggling ring sizes or heteroatom (re)placement, chemists can orchestrate “on-demand” reactivity that would be difficult to achieve through classical methods. Skeletal editing can also increase synthetic divergence and generate new analogs that were previously inaccessible or required lengthy synthetic steps. We hope this Account serves as an inspiration to generate more creative skeletal editing strategies and methods, and we look forward to seeing how this field continues to evolve especially with the aid of enzymatic catalysis, machine learning, and artificial intelligence.
